# Influence of serum vitamin D level on *Helicobacter pylori* eradication: A multi‐center, observational, prospective and cohort study

**DOI:** 10.1111/1751-2980.12793

**Published:** 2019-07-03

**Authors:** Chuan Han, Zhen Ni, Ting Yuan, Jian Zhang, Chan Wang, Xin Wang, Han Bing Ning, Jie Liu, Nina Sun, Cai Fang Liu, Miao Shi, Wen Quan Lu, Yong Quan Shi

**Affiliations:** ^1^ State Key Laboratory of Cancer Biology, National Clinical Research Center for Digestive Diseases, Xijing Hospital of Digestive Diseases Air Force Military Medical University Xi'an Shaanxi Province China; ^2^ Health Management Center Rocket Army Emei Sanatorium Emei Sichuan Province China; ^3^ Department of Gastroenterology General Hospital of the Western Theater Command Chengdu Sichuan Province China; ^4^ Department of Gastroenterology Hospital No. 150 of the People's Liberation Army Luoyang Henan Province China; ^5^ Department of Gastroenterology, Shaanxi Provincial People's Hospital Xi'an Shaanxi Province China; ^6^ Department of Gastroenterology Xianyang Central Hospital Xianyang Shaanxi Province China; ^7^ Department of Gastroenterology First Affiliated Hospital of Zhengzhou University Zhengzhou Henan Province China; ^8^ Xi'an Medical University Xi'an Shaanxi Province China

**Keywords:** eradication, *Helicobacter pylori*, infection, vitamin D

## Abstract

**Objectives:**

This study was designed to test whether serum vitamin D levels affected *Helicobacter pylori* (*H. pylori*) infection and eradication rates.

**Methods:**

A multicenter observational prospective cohort study was conducted. A total of 496 *H. pylori* ^−^ positive (*H. pylori* ^+^) and 257 *H. pylori*‐negative (*H. pylori* ^−^) patients were enrolled from four hospitals in China. Baseline serum vitamin D levels were measured and a ^13^C‐urea breath test (UBT) was performed for all the participants. The *H. pylori* ^+^ patients were divided into two subgroups based on their serum vitamin D levels (<10 or ≥10 ng/mL). A second ^13^C‐UBT was performed between 4 and 8 weeks after 14‐day bismuth‐containing quadruple eradication therapies. Factors potentially affecting *H. pylori* eradication were determined using a questionnaire survey.

**Results:**

Serum vitamin D levels were significantly lower in the *H. pylori* ^+^ group than in the *H. pylori* ^−^ group ([17.0 ± 6.9] ng/mL vs [19.2 ± 8.0] ng/mL, *P* = 0.000). *H. pylori* eradication rate significantly differed between patients with serum vitamin D levels of <10 ng/mL and ≥10 ng/mL (71.7% vs 87.3%, *P* = 0.005). A multivariate analysis showed that having serum vitamin D level ≥10 ng/mL was an independent risk factor for a successful *H. pylori* eradication (odds ratio 0.381, 95% confidence interval 0.183‐0.791, *P* = 0.010).

**Conclusions:**

Serum vitamin D level may affect *H. pylori* infection and its eradication. Randomized controlled trials are needed to find out whether vitamin D supplements may increase the *H. pylori* eradication rate.

## 1 INTRODUCTION

Gastric cancer (GC) generates a heavy disease burden in China. *Helicobacter pylori* (*H. pylori*) infection is known to play a role in the pathogenesis in most patients with GC.^1^ Accumulating evidence has shown that *H. pylori* infection causes almost 90% of non‐cardiac GC.^2^ Therefore, a successful eradication of *H. pylori* would significantly decrease the incidence of GC. However, both bacterial strains and host factors have been reported to impede the eradication of *H. pylori* infection, including the resistance to antibiotics, the virulence of the strains, and host‐related genetic disorders.^3^

Vitamin D regulates the calcium and phosphorus metabolism needed for bone formation, and the influence of vitamin D on *H. pylori* infection and eradication rates has recently been widely investigated. Several clinical studies have illustrated that vitamin D analogs may have anti‐*H. pylori* antimicrobial effects.^4^, ^5^, ^6^, ^7^, ^8^, ^9^, ^10^ Cytological research has also found that vitamin D_3_ decomposition product 1 (VDP1) can lyse *H. pylori* bacterial cells by inducing the collapse of the cell membrane.^11^, ^12^

However, the correlation between vitamin D levels and *H. pylori* has not been fully illustrated, and studies on the impact of serum vitamin D levels on *H. pylori* eradication were mostly of small sample sizes. Therefore, we conducted a multicenter prospective cohort study with a relatively large sample size to determine whether serum vitamin D levels had an impact on *H. pylori* infection and eradication, and whether its low level was an independent risk factor affecting *H. pylori* eradication.

## 2 PATIENTS AND METHODS

### 2.1 Study population

We conducted a non‐randomized, multicenter, observational and prospective cohort study with consecutively enrolled patients to estimate the influence of serum vitamin D levels on the rates of *H. pylori* infection and eradication. All patients were recruited between October 2017 and July 2018 from either one of the four centers: the Xijing Hospital of Digestive Diseases (Xi'an, Shaanxi Province, China), Xianyang Central Hospital (Xi'an, Shaanxi Province, China), the First Affiliated Hospital of Zhengzhou University (Zhengzhou, Henan Province, China), and the General Hospital of the Western Theater Command (Chengdu, Sichuan Province, China). Inclusion criteria were: (a) participants aged between 18 and 75 years; (b) having undergone a ^13^C‐urea breath test (UBT); and (c) those in whom the serum vitamin D level had been detected. The following individuals were excluded from the study: (a) aged less than 18 years old or over 75 years old; (b) had been treated for *H. pylori* infection; (b) had previously undergone gastric surgery; (d) pregnant or lactating; (e) had major systemic diseases; (f) had diseases that might affect their serum vitamin D levels, such as hyperthyroidism, malabsorption, rickets, osteoma, hypercortisolism, severe liver diseases (Child‐Pugh grade B or C), renal failure (serum creatinine >177 mmol/L) and alcoholism; (g) who had administered antimicrobial agents, bismuth agents or gastric antisecretory drugs during the previous 8 weeks; (h) had refused *H. pylori* eradication treatment, or were allergic to any one of the drugs given in the eradication schemes; and (i) who used daily vitamin D supplements. In total, 496 patients who were tested positive (*H. pylori* ^+^) and the 257 who were negative (*H. pylori* ^*−*^) for *H. pylori* infection and who had had their serum vitamin D levels tested were enrolled in our study. The study was approved by the Ethics Committees of the Xijing Hospital of the Air Force Military Medical University (no. KY20173035‐1), Xianyang Central Hospital (no. KY20171011‐1), the First Affiliated Hospital of Zhengzhou University (no. KY‐2017‐33), and the General Hospital of the Western Theater Command (no. KY20171023‐1). The study was conducted according to the ethical standards of the responsible committee on human experimentation (institutional and national) and the Helsinki Declaration (as revised in Brazil in 2013). Written, informed consent was obtained from all participants in the study.

### 2.2 Data collection

The following information were extracted from the patients who were *H. pylori* ^+^ using a questionnaire on the factors affecting *H. pylori* eradication, as follows: patients’ age, sex, occupation, residential area, body mass index (BMI), marital status, educational level, family members, annual income, smoking, alcohol consumption, history of periodontal disease, hygiene of dining place, main source of drinking water, drinking of untreated water or not, ^13^C‐UBT results, diagnosis by gastroscopy, treatment of adequate dosage and duration, choice of proton pump inhibitors (PPIs), and serum vitamin D levels. Treatment of an adequate dosage and duration was defined as having received one of two different *H. pylori* eradication schemes for 14 days without forgetting to take the prescribed medication.

### 2.3 Treatment and follow‐up for *H. pylori* ^+^ patients

The *H. pylori* ^+^ patients were further divided into two subgroups based on their serum vitamin D levels (<10 or ≥10 ng/mL) and were given one of the two treatment options for 14 days: (a) 1000 mg amoxicillin (Kelun Pharmaceutical Company, Chengdu, Sichuan Province, China) twice daily, 500 mg clarithromycin (Livzon Pharmaceutical Group, Zhuhai, Guangdong Province, China) twice daily, 220 mg colloidal bismuth tartrate capsule (Hunan Warrant Pharmaceutical Company, Changsha, Hunan Province, China) twice daily and 40 mg esomeprazole (AstraZeneca Pharmaceutical Company, Cambridge, UK) twice daily; or (b) 1000 mg amoxicillin (Kelun Pharmaceutical Company) twice daily, 500 mg clarithromycin (Livzon Pharmaceutical Group) twice daily, 220 mg colloidal bismuth tartrate capsule (Hunan Warrant Pharmaceutical Company) twice daily and 20 mg rabeprazole (Shanghai Pharmaceuticals Company, Shanghai, China) twice daily. The choice of *H. pylori* eradication scheme conformed to the Fifth Chinese national consensus report.^13^ A cut‐off value for serum vitamin D level was set at 10 ng/mL because a study from Turkey has reported that 10 ng/mL may be the cut‐off value that affects *H. pylori* eradication.^7^ Finally, a ^13^C‐UBT was repeated between 4 and 8 weeks after the treatment was completed.

### 2.4 Statistical analysis

Statistical analyses were performed using SPSS version 22.0 software (IBM, Armonk, NY, USA). A double entry and verification method was used to record and enter the data collected from the patients. A one‐sample Kolmogorov‐Smirnov test was adopted to test the normality of continuous variables, which were expressed as medians and ranges for the variables with an abnormal distribution and mean ± standard deviation for variables with a normal distribution. Comparisons between the groups were performed using the Student's *t*‐test, Wilcoxon signed‐rank test, or the χ^2^ test, when appropriate. A two‐tailed *P* < 0.05 was considered statistically significant.

Binary logistic regression was applied to determine whether an independent risk factor of *H. pylori* eradication was the patient's serum vitamin D level. The degrees of this association were measured using the odds ratio (OR) and 95% confidence interval (CI).

## 3 RESULTS

### 3.1 Patients' characteristics

**Figure 1:**
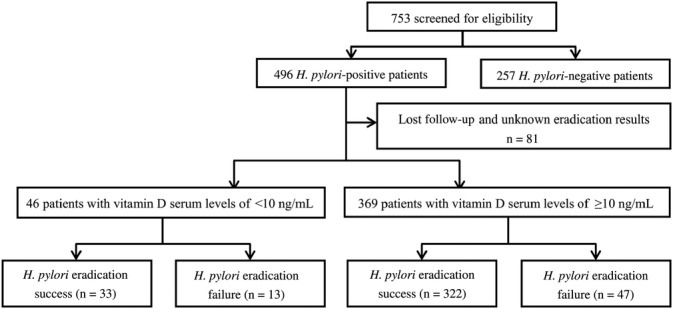
Flowchart of participant selection and grouping. *H. pylori*, *Helicobacter pylori*

As shown in Figure 1, there were 496 *H. pylori* ^+^ patients and 257 *H. pylori* ^*−*^ patients who underwent a ^13^C‐UBT and whose serum vitamin D levels were tested in our study. Of the *H. pylori* ^+^ patients, 81 (16.3%) out of 496 patients were lost to follow‐up; *H. pylori* was successfully eradicated in 355 (eradication rate 85.5% [355/415]).

### 3.2 Comparison of serum vitamin D levels between *H. pylori* ^+^ and *H. pylori* ^−^ patients

We found that serum vitamin D levels were significantly lower in the *H. pylori* ^+^ group than in the *H. pylori^−^* group ([17.0 ± 6.9] ng/mL vs [19.2 ± 8.0] ng/mL, *P* = 0.000); while there were no significant differences in others factors such as age, sex, season of vitamin D detection, or occupation between the two groups, as shown in Table 1. This indicates that serum vitamin D levels may affect *H. pylori* infection.

**Table 1:** Characteristics of the *Helicobacter pylori (H. pylori)*‐positive and ‐negative groups

Variables	*H. pylori*‐positive (N = 496)	*H. pylori*‐negative (N = 257)	*P* value
Age, y (mean ± SD)	47.1 ± 12.6	48.1 ± 10.2	0.259
Sex, n (male/female)	236/260	127/130	0.633
Season, n (spring/summer/autumn/winter)	266/38/25/167	144/15/12/86	0.794
Occupation, n (outdoor/indoor worker)	263/233	147/110	0.275
Vitamin D (25[OH]D), ng/mL (mean ± SD)	17.0 ± 6.9	19.2 ± 8.0	0.000

SD, standard deviation; 25(OH)D, 25‐hydroxyvitamin D.

### 3.3 Differences in the *H. pylori* eradication rate between patients with serum vitamin D levels <10 and ≥10 ng/mL

A second ^13^C‐UBT was performed between 4 and 8 weeks after 14‐day bismuth‐containing quadruple eradication therapy. Significant differences were seen between the serum vitamin D levels of <10 and ≥10 ng/mL groups in terms of their *H. pylori* eradication rates (71.7% vs 87.3%, *P* = 0.005; Table 2). This result suggests that having a lower serum level of vitamin D may be related to a lower eradication rate of *H. pylori*.

**Table 2:** Differences in *Helicobacter pylori* eradication rates between groups with serum vitamin D levels of <10 and ≥10 ng/mL

Vitamin D (25[OH]D)	Success (N = 355)	Failure (N = 60)	Eradication rate (%)	*P* value
<10 ng/mL	33	13	71.7	‐
≥10 ng/mL	322	47	87.3	0.005

25(OH)D, 25‐hydroxyvitamin D.

### 3.4 Multivariate analyses of factors affecting the *H. pylori* eradication rate

Factors influencing the eradication rate of *H. pylori* were analyzed using a questionnaire with 20 factors. As shown in Table 3, univariate analysis showed that a treatment of adequate dosage and duration, alcohol consumption and serum levels of vitamin D differed significantly between the groups in which *H. pylori* was successfully eradicated and that failed, suggesting that these factors might affect the *H. pylori* eradication rate.

**Table 3:** Factors affecting *Helicobacter pylori* eradication rates by univariate analysis

Variables	Success (N = 355)	Failure (N = 60)	*P* value
Sex, n (male/female)	162/193	32/28	0.269
Age, y (mean ± SD)	47.1 ± 12.7	48.2 ± 13.3	0.559
Occupation, n (outdoor/indoor worker)	164/191	31/29	0.432
BMI, kg/m^2^ (mean ± SD)	22.3 ± 3.2	21.8 ± 3.4	0.259
Residential area, n (urban/town/rural)	205/63/87	36/12/12	0.731
Marital status, n (single/married)	24/331	3/57	0.609
Education level, n (elementary school and below/middle school/university and above)	39/167/149	9/21/30	0.511
Family size, n (<4/≥4 people)	137/218	30/30	0.096
Annual income, n (<50 000/≥50 000 CNY)	198/157	35/25	0.712
Cigarette smoking, n (yes/no)	72/283	16/44	0.263
Alcohol consumption, n (yes/no)	23/332	9/51	**0.022**
History of periodontal disease, n (yes/no)	98/257	21/39	0.241
Hygiene of dining place, n (good/poor)	275/80	46/14	0.891
Main source of drinking water, n (clean/probably polluted)	307/48	56/4	0.138
Drinking untreated water, n (yes/no)	75/280	18/42	0.127
^13^C‐UBT value, n (Q1/Q2/Q3/Q4)*	91/83/88/93	11/22/16/11	0.671
Diagnosis by gastroscopy, n (PU/CNAG/CAG/IM/GED)	15/180/142/16/2	3/27/29/1/0	0.623
Treatment with adequate dosage and duration, n (yes/no)	324/31	44/16	**0.000**
Choice of PPI, n (esomeprazole/rabeprazole)	177/178	35/25	0.225
Vitamin D serum level, n (<10/≥10 ng/mL)	33/322	13/47	**0.005**

*Note*: *^13^C‐urea breath test (UBT) values were distributed according to quartile: Q1, P_0_‐P_25_; Q2, P_25_‐P_50_; Q3, P_50_‐P_75_; and Q4, P_75_‐P_100_. P_25_ = 9.8; P_50_ = 20.3; and P_75_ = 35.6. Statistically significant values are bold.

Abbreviations: BMI, body mass index; CAG, chronic atrophic gastritis; CNAG, chronic non‐atrophic gastritis; GED, gastric epithelial dysplasia; IM, intestinal metaplasia; PPI, proton pump inhibitor; PU, peptic ulcer; SD, standard deviation.

A further multivariate analysis revealed that an adequate dosage and duration of treatment (OR 0.265, 95% CI 0.133‐0.530, *P* = 0.000) and serum vitamin D levels of ≥10 ng/mL (OR 0.381, 95% CI 0.183‐0.791, *P* = 0.010) were independent risk factors for a successful *H. pylori* eradication (Table 4).

**Table 4:** Factors affecting *Helicobacter pylori* eradication rate by multivariate analyses

Variables	OR (95% CI)	*P* value
Serum vitamin D level (≥10/<10 ng/mL)	0.381 (0.183‐0.791)	**0.010**
Treatment with adequate dosage and duration (yes/no)	0.265 (0.133‐0.530)	**0.000**
Alcohol consumption (no/yes)	0.821 (0.292‐2.273)	0.704

*Note*: Statistically significant values are in bold. Abbreviations: CI, confidence interval; OR, odds ratio.

## 4 DISCUSSION

Since the discovery of *H. pylori* in 1982 its related diseases have been studied worldwide. In 1994 the International Agency for Research on Cancer identified *H. pylori* as a class I carcinogen.^14^ In 1997 the Maastricht consensus produced a world agreement on *H. pylori* infection. Since then the Consensus has been modified four times,^15^, ^16^, ^17^, ^18^, ^19^ and our understanding of the role of *H. pylori* in GC has been constantly updated. The occurrence of GC was used to be connected to a combination of *H. pylori* infection, host factors and environmental factors. However, currently 90% of non‐cardiac GC are related to *H. pylori* infection,^2^, ^20^ the overall function of environmental factors in GC has been found to be subordinate to that of *H. pylori* infection, and genetic factors are considered truly decisive in only 1%‐3% of hereditary diffuse GC.^21^ According to the latest consensus, the eradication of *H. pylori* should be a primary preventative measure for GC, as *H. pylori* infection is currently the foremost controllable risk factor for GC.^19^, ^22^, ^23^

China has a high level of *H. pylori* infection rate, with an overall infection rate of 56.22%, and Tibet even has the highest infection rates worldwide of 84.62%.^24^ Bacterial and host factors that need to be taken into account in *H. pylori* eradication include antibiotic resistance, virulence of the strains and host‐related genetic disorders.^3^ However, the drug resistance rates of *H. pylori* to clarithromycin, metronidazole and levofloxacin (fluoroquinolones) in China have been rising. In recent years, drug resistance rates of *H. pylori* have been reported to be 20%‐50% to clarithromycin, 40%‐70% to metronidazole and 20%‐50% to levofloxacin.^25^, ^26^, ^27^, ^28^, ^29^, ^30^, ^31^, ^32^ *H. pylori* can develop double, triple, or even greater resistance to these antibiotics.^25^, ^26^, ^27^ The reported double resistance rate to clarithromycin and metronidazole is more than 25%.^28^, ^29^, ^30^ Moreover, the type of *H. pylori* strain also affects its eradication. *H. pylori* contains a variety of virulent factors, including cytotoxin‐associated gene A, vacuolating cytotoxin A, duodenal ulcer promoting gene, outer inflammatory protein A and blood group antigen‐binding adhesion, which affect gastric mucosal inflammation and injury by activating inflammatory cell infiltration. Because virulence factors play a crucial part in gastric mucosal injury and affect *H. pylori* therapy,^33^ the virulence of different strains affects the eradication of *H. pylori* infection. In addition, the successful treatment of *H. pylori* infection also depends on genetic factors of the host, such as cytochrome P450 2C19, interleukin‐1β, and multidrug resistance gene 1.

Vitamin D is responsible for regulating calcium and phosphorus metabolism, which are needed for bone formation; however, many recent studies have found that vitamin D also affects *H. pylori* eradication. One study showed that VDP1 had a selective bactericidal effect on *H. pylori* but made no difference to the viability of Enterobacteriaceae bacteria, *Pseudomonas aeruginosa*, or *Staphylococcus aureus*.^11^ Studies have suggested that dimyristoylphosphatidylethanolamine (DMPE) is one of the most common glycerophospholipids constituting the cell membrane of *H. pylori*,^34^, ^35^ and VDP1 may induce bacterial dissolution by interacting with the DMPE of the *H. pylori* membrane. Another study found that the alkyl of indene, a product of vitamin D decomposition, had the key conformation of interaction with the di‐14:0 DMPE in the lipid membrane component of *H. pylori*, which eventually induced lysis, and the absence of alkyl led to the loss of a bactericidal effect on *H. pylori*.^12^ Several clinical studies have illustrated that vitamin D analogs may have anti‐*H. pylori* antimicrobial effects.^4^, ^5^, ^6^, ^7^, ^8^, ^9^, ^10^ Some studies have also found that serum vitamin D levels may have an impact on *H. pylori* eradication.^7^, ^8^, ^9^

However, the correlation between vitamin D and *H. pylori* remains unknown, and studies of the impact of serum vitamin D levels on *H. pylori* eradication included small sample sizes. Thus, we conducted this multicenter observational prospective cohort study to determine whether serum vitamin D levels had an impact on *H. pylori* infection and eradication rates and whether this was an independent risk factor for *H. pylori* eradication. A total of 496 *H. pylori* ^+^ and 257 *H. pylori* ^−^ patients were enrolled from four centers, and the patient population were from most areas of Mainland China. The results showed that serum vitamin D levels were lower in the *H. pylori* ^+^ group than in the *H. pylori* ^*‐*^ group. Furthermore, a low level of serum vitamin D in patients was related to a reduced *H. pylori* eradication rate. A multivariate regression analysis of the collected factors influencing *H. pylori* eradication demonstrated that serum vitamin D level <10 ng/mL was an independent risk factor for the failure to eradicate *H. pylori*. However, the notion that vitamin D supplements might decrease the infection rate of *H. pylori* and increase its eradication rate has yet to be evaluated. Unfortunately, we were unable to calculate the sample size needed due to the lack of specific data about the influence of serum vitamin D levels on the *H. pylori* eradication rate, although our sample size was relatively large. Therefore, randomized controlled studies with large sample sizes are urgently needed to validate our findings and to clarify whether vitamin D supplements improve the eradication rate of *H. pylori*, in order to provide guidance for *H. pylori* eradication therapy and the prevention of GC.

## 5 CONFLICT OF INTEREST

None.
